# ERP evidence for consumer evaluation of copycat brands

**DOI:** 10.1371/journal.pone.0191475

**Published:** 2018-02-21

**Authors:** Qian Shang, Guanxiong Pei, Jia Jin, Wuke Zhang, Yuran Wang, Xiaoyi Wang

**Affiliations:** 1 Chinese Academy of Science and Education Evaluation, Hangzhou Dianzi University, Hangzhou, China; 2 Management School, Hangzhou Dianzi University, Hangzhou, China; 3 School of Management, Zhejiang University, Hangzhou, China; 4 Business School, Ningbo University, Ningbo, China; 5 Academy of Neuroeconomics and Neuromanagement at Ningbo University, Ningbo, China; University of Pennsylvania, UNITED STATES

## Abstract

Copycat brands mimic brand leaders to free ride on the latter's equity. However, little is known regarding if and how consumers confuse copycat as leading brand in purchasing. In this study, we applied a word-pair evaluation paradigm in which the first word was a brand name (*copycat vs*. *normal brand both similar with a leading brand in category*), followed by a product name (*near vs*. *far from the leading brand’s category*). Behavioral results showed that, when the product is near the leader’s category, the copycat strategy (CN) was more preferred compared to the normal brand (NN) but not different in the far product condition (CF and NF). Event-related potential (ERP) data provided further insight into the mechanism. The N400 amplitude elicited by the CN condition was significantly smaller than NN. However, when products are far from the leader’s category, there was no significant difference in N400 amplitudes. For the late positive component (LPC), the CN gave rise to a larger amplitude than the CF. The N400 amplitude was suggested to reflect the categorization process, and the LPC demonstrated the recollection process in long-term memory. These findings imply that the copycat brand strategy is generally only effective when products are within the category of the leading brand, which offers important implications for marketing practices.

## Introduction

Brand perception is an important issue and a special phenomenon of social cognition that is prevalent in daily life. A brand can be regarded as a mental category of products, which helps consumers to distinguish the product of one company from that of another [1]. Thus, the brand is regarded as an intangible asset, which can often be the most valuable asset on a corporation’s balance sheet. Most brands require a long time and significant efforts to build. Firms invest significant resources in advertising and packaging [2,3] to maintain positive associations with their products. Merchants eager for quick success and instant benefits may seek shortcuts to increase sales, and thus copycat brands were created. Copycat brands are defined as brands that imitate the features of well-known brands to obtain a “free ride” on their high brand equity [4]. In marketing practices, copycat brands can imitate the names of leading brands to induce perceptual similarity legitimately. Look-alike copycats imitate various features such as the spellings of leading brand names by replacing or rearranging one or more letters of the brand name; for instance, Aldi’s Norpak spreadable butter brand name looks similar to that of the leading brand, Lurpak [5].

Although copycatting is currently a widespread branding strategy [6], evaluations of copycat brands remain elusive, and existing results are controversial. Some studies shows that copycat brands leaded to more positive evaluations by consumers [7]. Nevertheless, completely opposing viewpoints have also been reported regarding copycat brand evaluation for its giving rise to negative feelings because of counterfeiting motivies [8]. Even for some products, a low-similarity copycat brand name was preferred over an identical brand name [9]. These copycat activities may result in confusion for consumers [10]. The underlying mechanism of this effect can be traced to consumer persuasion knowledge. Simple imitation activates consumer persuasion knowledge regarding the insincere motives of the copycat brand, which in turn shapes their brand evaluations [5]. Thus, one focus of this study was the comparison of evaluations of copycat brand names (similar to the leading brand) and normal brand names (dissimilar to the leading brand).

A second and more specific focus of this study was the relationship of the product category with copycat brands. A copycat branded product involves the use of a copycat brand name to launch new products. There are two types of copycat branded product strategies: near copycat branded products within the category of the leading brand and far copycat branded products that are outside the category of the leading brand. To our knowledge, no systematic empirical study has addressed the question of how product categories that are the same or different from that of the leading brand influence the evaluation of copycat brands, namely, the effects of copycat branded products near the leading brand’s category (CN) and copycat branded products far from the leading brand’s category (CF).

The aim of this study was to reveal the mechanism of copycat branded product evaluation. Previous studies on copycat brands have mainly relied on self-reporting measurement scales [5,6]. Using self-reported measurements, people may not be able to fully articulate their preferences when asked to express themselves explicitly, and furthermore, consumers often have hidden feelings in the brain regarding their true preferences [11]. In some cases, people are unwilling to state their actual thoughts and preferences through verbal or written self-reports [12,13]. As a result, the use of self-reported measurements to evaluate people’s attitudes, preferences or purchase intent of the copycat might result in observable biases and large variance. To overcome these limitations of self-reporting, we utilized a novel emerging technique based on event-related potentials (ERP), which were used to directly access consumers’ mental activities underlying their observed behaviors [14]. Examining brain activity while subjects perform behavior tasks can help deeply evaluate how people perceive and process information and make decisions [15]. Moreover, ERPs have a high temporal resolution and, therefore, can capture the real-time processes and cognitive mechanisms that underlie observed behaviors, which cannot be obtained through traditional methods [16]. Thus, ERPs have been widely used to study cognitive processes in brand perception and brand extension evaluation [17,18]. The current study used ERPs to evaluate copycat brand perception and compared product category strategies at the level of brain activity by recording electroencephalograms (EEGs) throughout the experiment. To fulfil the aim of this study, we sought to identify ERP components N400 and LPC that can be both appropriate and reliable for measuring copycat brand perception and product category evaluation.

The N400 component is a negative-going deflection that peaks approximately 400 ms post-stimulus onset (for a recent review, see [19]). Many studies have indicated that the N400 component can be considered as an index of categorization processing for semantic meaning [20–23]. For example, Rugg (1985) found that stimuli that violate semantic categories (e.g., “I have classes with my door”) elicit a larger N400 component than stimuli in congruent semantic categories (e.g., “I have classes with my classmates”) [20]. Heinze and colleagues (1998) demonstrated that the N400 component could be deemed as an index of category membership, and typical exemplars of a category yielded smaller N400 amplitudes than atypical entities [21]. When participants were required to evaluate the accuracy of simple sentences about category membership (e.g., ‘a carrot is a vegetable’), a reduced N400 amplitude was found for exemplars belonging to the category [22]. In a picture-word matching task that presented the word pairs “CAMEL”–“camel” (exact match), “CAMEL”–“cow” (in category), and “CAMEL”–“candle” (out of category), out of category words elicited a larger N400 component than other pairs of words [23]. Furthermore, a series of recent studies have examined the neurophysiological processes of brand extension with a prime-probe paradigm and found that N400 reflects the categorization association between leading brands and extension products [17,18,24]. For instance, Wang et al. (2012) compared two types of words in brand-product pairs (matching vs mismatch in category). The N400 was enlarged in the mismatch condition, indicating a larger cognitive reaction when the product’s attributes were atypical of the category of the brand [24]. A further study of brand-product evaluation by Ma et al. (2014) showed that N400 could distinguish subcategory products from major-category products, and it was regarded as the second stage of the categorization process in evaluation [17]. Previous research has also demonstrated that a closer association between brand name and product name can result in smaller N400 amplitudes [18]. Therefore, we hypothesized that a copycat brand for a near product category would elicit a smaller N400 amplitude than a normal brand because of the close association between the copycat brand and the typical product of the leading brand. However, compared with the normal brand, the copycat brand for a far product category would not elicit a significantly different N400 amplitude because the far product could not be looked as the typical member of neither copycat nor normal brand.

The late positive component (LPC) is a slow centro-parietal positive ERP wave elicited between 500 and 800 ms after a stimulus, which is closely associated with familiarity and recollection [25–27]. Previous studies have reported that successful recognition of old words gives rise to the LPC [28,29]. LPC amplitudes are larger for *Remember* responses than for merely *Know* responses [30–32]. In an old/new recognition experiment by Finnigan et al. (2002), the LPC was found to be sensitive to the accuracy of recognition decision, and its amplitude was larger in correct recognition versus incorrect recognition [33]. Gutchess et al. (2007) showed that the recognition effect of LPC was larger when participants were highly confident in their ‘old’ judgements than when they were less confident [34]. LPC enhancement has also been reported for familiar compared with unfamiliar stimuli [27]. In a study by Voss and Paller (2008), when participants were asked to recognize a repeated squiggle as either meaningful or meaningless, LPC potentials were found to be associated with familiarity ratings during recognition irrespective of the level of meaningfulness [35]. In this study, we hypothesized that copycat brands combined with near products would elicit a larger LPC than copycat brands combined with far products because copycat branded near product would more easily active the association of the leading brand and its products. In addition, we supposed that LPC amplitude would be smaller in the copycat brand condition than in the normal brand condition when the product category was a far product, because the copycat brand here could not arouse the retrieval of the imitated leading brand and might be looked as an unknown new brand, which mean it could more hardly active the memory recollection process than a normal brand. This process was strongly linked to explicit reactivation of stored memory traces, and LPCs were sensitive to memory recollection.

## Methods

### 2.1. Subjects

Twenty undergraduate students (11 males, mean age = 21.23 years, SD = 1.06) from a large university were recruited for this study as paid volunteers. All the volunteers were right-handed and had normal or corrected-to-normal vision. No participants reported a history of neurological disorders or mental disease. The study was approved by Ethics Committee of Neuromanagement Study at Zhejiang university of China. Written informed consent forms were obtained from all subjects before beginning the experiment.

### 2.2. Materials

In this experiment, the size of each stimulus was 300 × 400 pixels. Target stimuli were 208 pairs of brand name-product name, which comprised 52 brand names and 52 product names. There were two groups of brand names. One group (26 brand names) included copycat brands (e.g., Cock-cola) created from well-known leading brands correspondingly (e.g., Coca-Cola). The other group (26 brand names) included normal brands in a category similar to that of the leading brand (e.g., Feichan-cola, a local brand in East China). The product names comprised the two following groups: near products in the leading brand category (26 product names, e.g., ice tea) and far products outside the leading brand category (26 product names, e.g., sushi). Therefore, there were four stimulus conditions (2 brand strategies × 2 product categories), as follows:

1. CN: a copycat brand paired with a product within the category (near product) of the leading brand, such as Cock cola-ice tea, Aoudi-motorcycle;
2. CF: a copycat brand paired with a product outside the category (far product) of the leading brand, such as Cock cola-sushi, Aoudi-battery;
3. NN: a normal brand in the category of the leading brand paired with a product within the category (near product) of the leading brand, such as Feichan-ice tea, Geely-motorcycle;
4. NF: a normal brand in the category of the leading brand paired with a product outside the category (far product) of the leading brand, such as Feichan-sushi, Geely-battery.

For all stimuli, a pretest was performed to evaluate familiarity (“Have you seen the brand name before?”) and product distance (“How far is the XX product to the leading brand XX”) by using 7-point items, and 83 valid data points were collected. The ANOVA results demonstrated no significant main effects of brand strategy [*F*(1,82) = 0.027, *p* = 0.87, *η*^*2*^ = 0.01] but significant difference between near and far products [*F*(1,82) = 10.82, *p*<0.05, *η*^*2*^ = 0.36] (M = 1.60, S.E. = 0.67 vs M = 4.56, S.E. = 1.21).

### 2.3. Procedure

**Fig 1:**
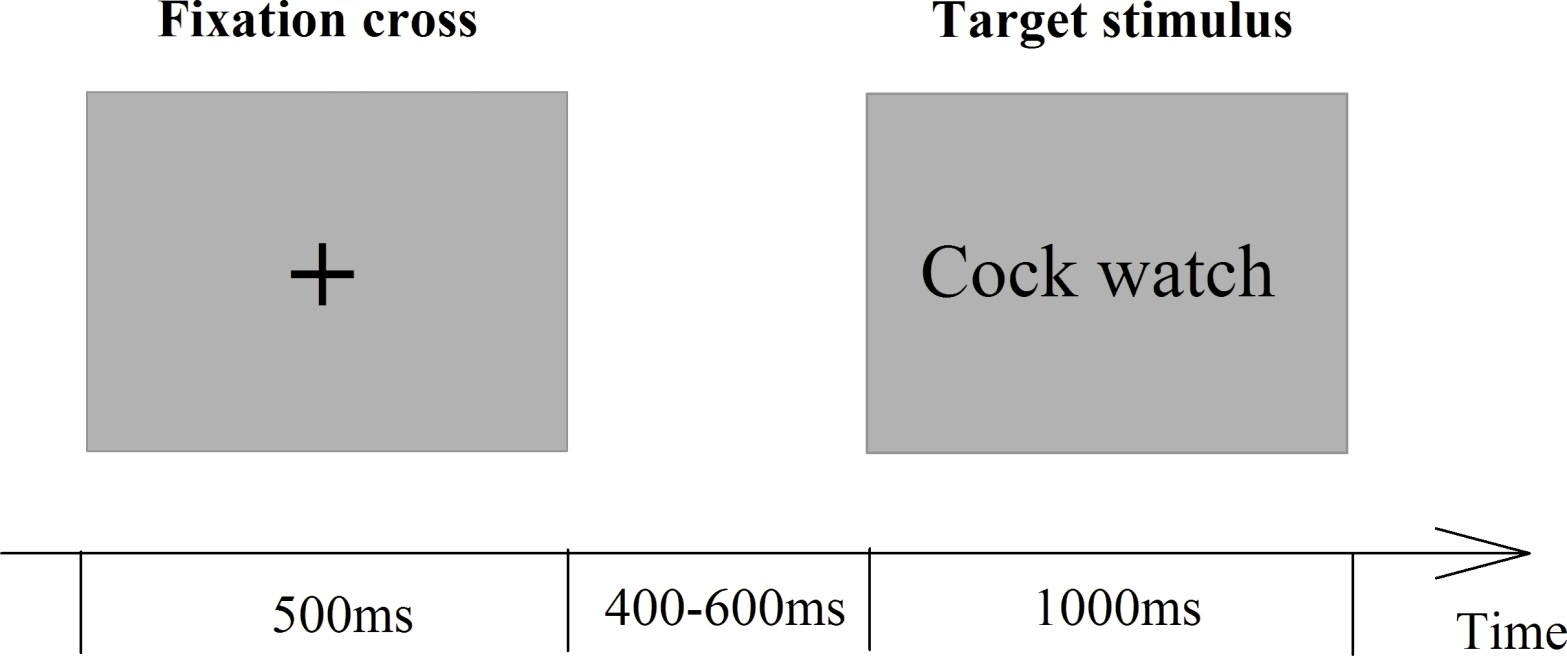
Experimental procedure. The participants were presented with four brand-product strategies [CN, CF, NN and NF]. They were instructed to complete the brand-product evaluation tasks and had a maximum of 1500 ms to make each choice. EEGs of the subjects were recorded throughout the experiment.

All stimuli were divided into 4 blocks of 208 trials each. The formal experiment started after 20 practice trials. A stimulus was presented in the center of a computer screen using a stimulus system (Stim2, Neurosoft Labs, Inc., Sterling, VA, USA) 90 cm in front of the subjects. Subjects were provided a keypad to respond. In each trial (see Fig 1), a central fixation cross appeared for 500 ms, followed by a target stimulus, which was presented for a duration of 1000 ms. The subjects were instructed to evaluate whether they would like the presented brand-product pair if it was being sold in the marketplace. These brand-product pairs were randomized by the program to prevent the subject from predicting the upcoming task. The subjects had a maximum of 1500 ms to respond by pressing a button. The response buttons were counterbalanced across subjects.

### 2.4. Electroencephalogram (EEG) recording and analysis

A set of 64 Ag/AgCl electrodes was used for EEG recordings (bandpass 0.05–100 Hz, sampling rate 500 Hz) with a Neuroscan Synamp2 Amplifier (Scan 4.3.1, Neurosoft Labs, Inc. Virginia, USA). A cephalic electrode was used applied as the ground. The left mastoid served as the on-line reference, and the average of the left and right mastoids was used as the off-line reference. Electrooculograms (EOG) were recorded with two pairs of electrodes. One pair was used to record vertical EOG, which was placed on the supra and infra-orbital locations of the left eye, while the other pair was used to record horizontal EOG, which was placed 10 mm from the lateral canthi of both eyes. Electrode impedances were maintained below 5 kΩ during the experiment. During the offline EEG analysis, electrooculogram artifacts were corrected using the method proposed by Semlitsch et al. (1986) [36]. EEG recordings were segmented into time-locked -200 to 800 ms epochs relative to the onset of the target stimulus, with the prestimulus period used as baseline. Trials containing amplifier clipping, bursts of electromyography activity, or peak-to-peak deflection exceeding ± 80 μV were excluded. The ERP waveforms were averaged for every participant in four conditions (CN, CF, NN and NF). More than 50 sweeps for each condition remained, which were adequate to achieve stable and reliable measurements of the N400 component and the LPC [37]. The averaged ERP waveforms were digitally filtered with a low-pass 30-Hz filter (24 dB/octave).

Based on visual observation of the grand average waveforms, we averaged the ERP amplitudes of the 250–450 ms time window for the N400 component and the 500–650 ms time window for the LPC component. According to previous studies and the topographic distribution of corresponding components, the F3, Fz, F4, FC3, FCz, FC4, C3, Cz and C4 electrodes were selected for the N400 component, and the C3, Cz, C4, CP3, CPz, CP4, P3, Pz and P4 electrodes were selected for the LPC in the statistical analysis. To study the neurophysiological features of the evaluation process on different brand-product strategies, a within-subjects repeated measures ANOVA corresponding to 2 *brand strategies* (copycat brand/normal brand) × 2 *product categories* (near product/far product) × 9 *electrodes* was conducted for the N400 component and the LPC. Greenhouse–Geisser [38] correction was applied when necessary, and the Bonferroni correction was used for multiple paired comparisons.

## Results

### 3.1. Behavioral results

For behavioral data, ANOVA was used to analyze the acceptance rate. The acceptance rate referred to the rate of *like* evaluations reported by the subjects.

**Fig 2:**
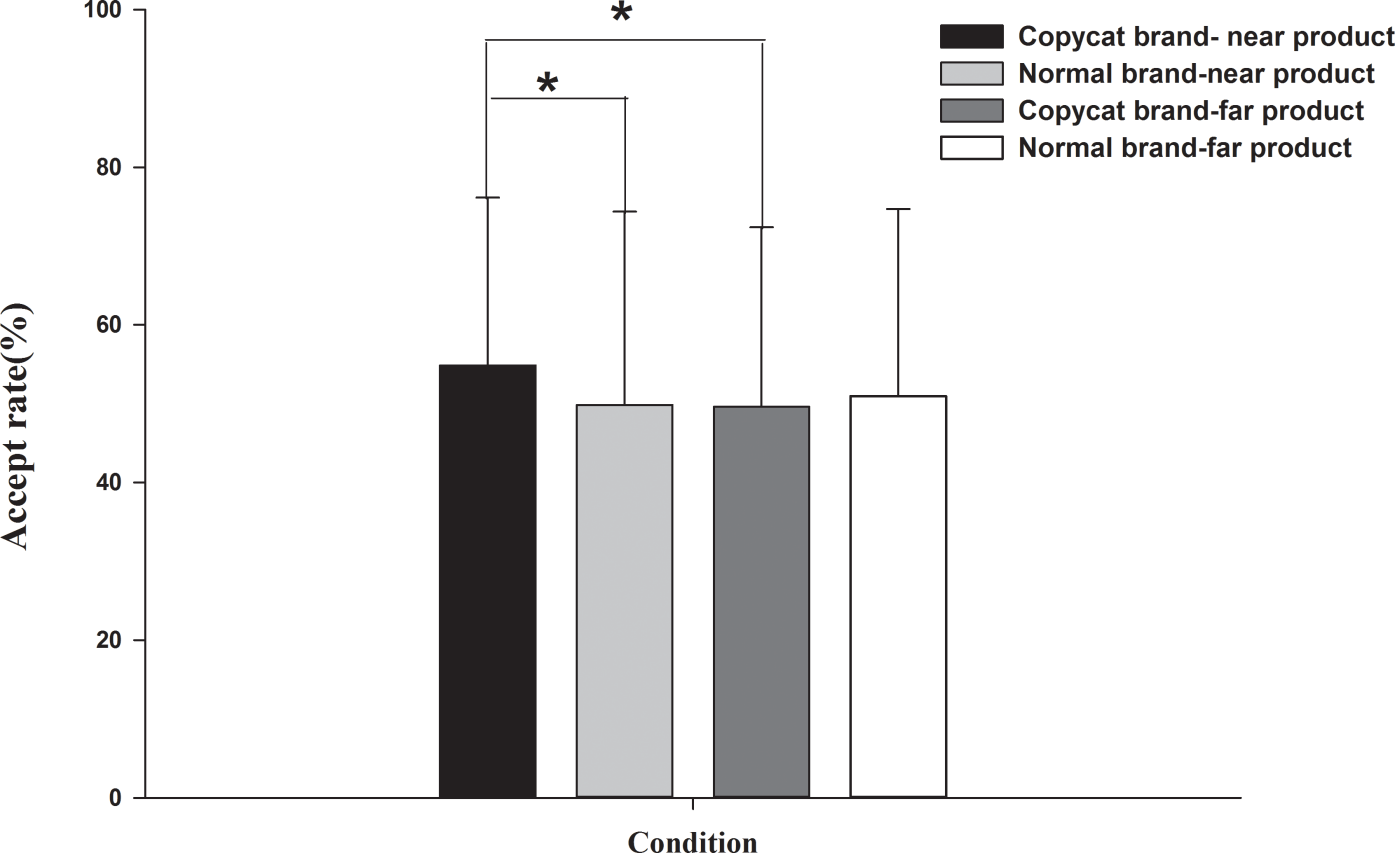
Behavioral results. Acceptance rates of the four brand-product strategies [Copycat brand-near product, Copycat brand-far product, Normal brand-near product and Normal brand-far product].

For acceptance rate, there was a significant interaction effect between *brand strategy* and *product category* [*F*_(1,19)_ = 6.26, *p*<0.05, *η*^2^ = 0.25]. Further analysis of the simple effect revealed that the copycat brand (CN: *M* = 55%, S.E. = 5%) had a larger acceptance rate than the normal brand (NN: *M* = 49%, S.E. = 6%) only for near product as the product category [*F*_(1,19)_ = 7.33, *p*<0.05]. Additionally, the CN condition (*M* = 55%, S.E. = 5%) showed a larger acceptance rate than the CF condition (*M* = 49%, S.E. = 5%) [*F*_(1,19)_ = 5.85, *p*<0.05] (see Fig 2). The main effects of *brand strategy* [*F*_(1,19)_ = 1.21, *p*>0.05, *η*^2^ = 0.06] and *product category* [*F*_(1,19)_ = 1.78, *p*>0.05, *η*^2^ = 0.09] were not significant.

### 3.2. ERP results

**Fig 3:**
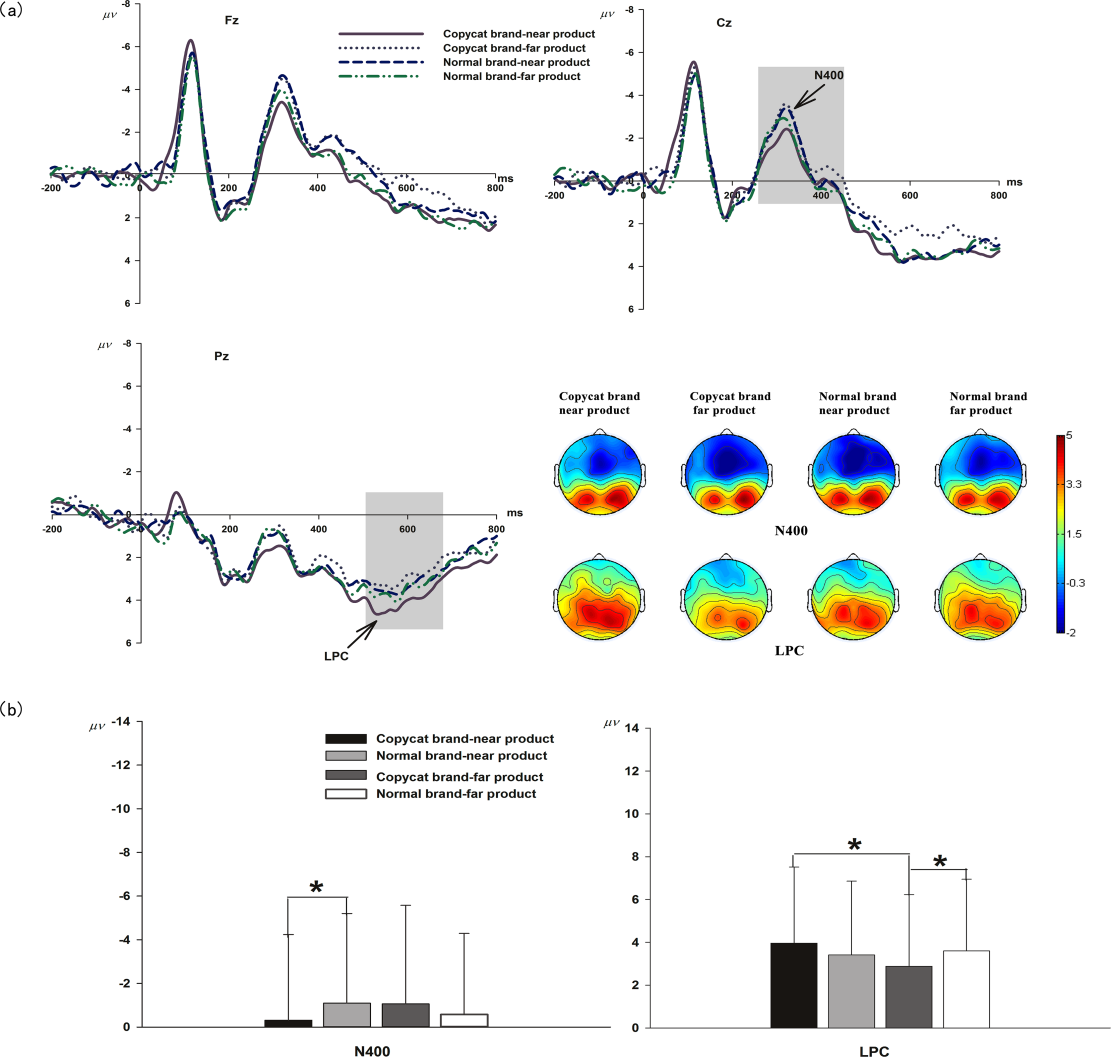
ERP results. (a) Grand averaged ERP of the N400 component and the LPC elicited by the four brand-product strategies [Copycat brand-near product, Copycat brand-far product, Normal brand-near product and Normal brand-far product] from 3 midline electrodes in the forehead, central and parietal areas [Fz, Cz, and Pz]. The scalp topographic distributions of the N400 amplitudes and LPCs are provided; scale bar of the topographic map ranges from −2 to 5 μV. (b) Average amplitudes of the N400 component and the LPC component in four brand-product strategies.

As presented in Fig 3, for N400 amplitude, ANOVA revealed a significant main effect of *electrode* [*F*_(8, 152)_ = 3.69, *p*<0.01, *η*^2^ = 0.16] and an interaction effect between *brand strategy* and *product category* [*F*_(1, 19)_ = 5.34, *p*<0.05, *η*^2^ = 0.22]. Simple effect analysis revealed that the difference in N400 amplitude between the copycat brand (CN: *M* = -0.32 μV, SE = 0.88) and the normal brand (NN: *M* = -1.09 μV, SE = 0.92) was only significant for the near product category [*F*_(1,19)_ = 6.82, *p*<0.05]. There were no significant main effects of *brand strategy* [*F*_(1,19)_ = 0.30, *p*>0.05, *η*^2^ = 0.02] and *product category* [*F*_(1,19)_ = 0.29, *p*>0.05, *η*^2^ = 0.02] and no interaction effects between *brand strategy* and *electrode* [*F*_(8, 152)_ = 0.84, *p*>0.05, *η*^2^ = 0.04], between *product category* and *electrode* [*F*_(8, 152)_ = 0.49, *p*>0.05, *η*^2^ = 0.03], or between *brand strategy*, *product category* and *electrode* [*F*_(8, 152)_ = 0.79, *p*>0.05, *η*^2^ = 0.04].

For the LPC component, ANOVA revealed a significant main effect of *product category* [*F*_(1,19)_ = 4.78, *p*<0.05, *η*^2^ = 0.20] and an interaction effect between *brand strategy* and *product category* [*F*_(1,19)_ = 9.27, *p*<0.01, *η*^2^ = 0.33]. Simple effect analysis revealed a significant LPC difference between the copycat brand (CF: *M* = 2.88 μV, SE = 0.75) and the normal brand (NF: *M* = 3.60 μV, SE = 0.75) for the far product category [*F*_(1,19)_ = 5.00, *p*<0.05]. This analysis also revealed that the mean LPC amplitude of the near product category (CN: *M* = 3.96 μV, SE = 0.80) was significantly larger than the mean LPC amplitude of the far product category (CF: *M* = 2.88 μV, SE = 0.75) in the copycat brand strategy [*F*_(1,19)_ = 4.78, *p*<0.05]. The main effects of *brand strategy* [*F*_(1,19)_ = 0.12, *p*>0.05, *η*^2^ = 0.01] and *electrode* [*F*_(8, 152)_ = 1.13, *p*>0.05, *η*^2^ = 0.06] and the interaction effects between *brand strategy* and *electrode* [*F*_(8, 152)_ = 0.69, *p*>0.05, *η*^2^ = 0.04], between *product category* and *electrode* [*F*_(8, 152)_ = 0.23, *p*>0.05, *η*^2^ = 0.01], and between *brand strategy*, *product category* and *electrode* [*F*_(8, 152)_ = 1.30, *p*>0.05, *η*^2^ = 0.06] were not significant.

## Discussion

This study explored the neural evidence of copycat brand evaluations, integrating the effects of brand similarity and product category. To the best of our knowledge, this is the first study to use ERP to investigate specific brain activity related to copycat brand perception and product category evaluation. Both ERPs and behavioral results indicate that copycat effect will disappear when its product is far from the leading brand’s category.

Regarding behavioral results, the copycat had a larger acceptance rate than the normal branded product in near condition but no difference in far condition. Furthermore, we found that the acceptance rate in the CN condition was higher than in the CF condition. These findings might be attributed to a perception fluency theory, which suggests that the linguistic similarity of a well-known or previously presented stimuli can result in more fluent processing in evaluation [39,40]. Based on this perspective, the CN strategy, which activated associations with the memories of pleasant experience of the leading brand and its products, could encourage consumers to feel more fluent compared to other strategies. Thus, the positive evaluations associated with the leader brand are likely to be infused into the copycat brand [10,41], giving rise to the positive evaluations observed for the CN strategy.

However, our results may conflict with the results of some previous studies, which suggested that copycat activities were not preferred by consumers[5,8,9]. For instance, Horen and Pieters (2012) indicated that high-similarity copycat brands were evaluated more poorly compared to moderate-similarity copycats [8]. In a study by Qin et al. (2016), copycat brands that simply imitated perceptual features of a leading brand received negative evaluations [5]. In these studies, evaluation comparisons were conducted among different similarity levels of copycat brands. The underlying mechanism of the poor evaluations received by high-similarity copycats was traced to consumer persuasion knowledge, which refers to peoples’ perceptions and beliefs about marketers’ motives and manipulative intents [5,42]. Persuasion knowledge regarding insincere motives of high-similarity copycat brands, in turn, negatively shapes brand evaluations [5]. However, in the current study, we observed that copycat brands had an advantage compared to normal brands when their familiarties were controlled. It may be that people did not perceive an obvious imitation activity of the copycat brand when it was compared to an normal brand that was independent of the leading brand, and hence, persuasion knowledge was not activated. This deduction could be supported by a previous study, which demonstrated that noncomparative evaluations (such as when the leader brand is not presented) are likely to be more beneficial to high-similarity copycats than to moderate- and low-similarity copycats [8].

In terms of ERP components, the N400 component could be considered to reflect the cognitive categorization process [17,18,20,21,23,24]. As elaborated in the Introduction, brand evaluation studies have reported that the N400 amplitude is larger in the out-of-category extension condition than in the in-category extension condition, which indicates a larger cognitive reaction when the target’s attributes are atypical of the category of the prime[17,24]. The N400 component can describe associations between word pairs, such as the brand name and the product name [18]. N400 could also reflect the activated condition induced by the current input stimuli in the long-term memory system. The N400 amplitude is smaller for stimuli that are similar or related to things stored in memory [19,43]. In this study, the CN condition elicited a lower N400 amplitude than the NN condition. It is possible that the copycat brand-near product combination activated association of the leading brand and the following near product which was more possibly looked as its typical product than primed with normal brand. This copycat effect led to a lower N400 component. However, this category association effect was only effective for near products. When the product category was far from the leading branded product category, neither the copycat branded product (CF) nor the normal branded product (NF) could be looked as the typical exemplar of the brand. As a result, we found no significant difference in N400 amplitudes between the CF and NF conditions. Thus, the N400 component here could be considered a reflection of the categorization process with which a near product was more easily looked as a member of the copycat brand compared with the normal branding strategy.

In addition to N400, the emergence of a LPC was also observed in the present study. The LPC has been closely associated with memory recollection [25–27,34,44]. Successful recognition of a thing gives rise to LPC [27–33,35]. In this experiment, we found a larger LPC amplitude in copycat branded near product than that in copycat branded far product. It is possible that, copycat brands combined with near products than far products could more easily active the association of the imitated leading brand and its products. This familiar association triggered the memory recollection process and then contributed to a larger LPC component in the CN condition. This finding suggested that the copycat strategy was effective in the memory recollection stage when the copycat brands were combined with near products. However, interestingly, we found that LPC amplitude was significantly smaller in the CF condition than in the NF condition. It may be that copycat brands combined with far products could not arouse the retrieval of the imitated leading brand. Therefore, the copycat effect in the memory recollection stage was blocked. It means when the far product condition, the copycat brand might be looked as an unknown new brand and could not obtain the “free ride” of the imitated leading brand. As a result, in the far procuct condition, a copycat brand that was looked as an unknown new brand could more hardly active the memory recollection process than a normal brand, thus leading to lower LPC amplitudes. In the cognitive stage, the LPC component here was suggested as a reflection of the memory recollection process.

A company generally must spend $50 million to $100 million to fully introduce a new brand name to the market [45]. Copying the names of leading brands can reduce the commensurate investments and costs of brand building. In East Asian developing countries, this phenomenon is prevalent and is particularly used to ride on the high brand equity of multinational brands [5]. According to the present study, copycat branding is useful for promoting new products only when the new products are near the category of the leading brand. Thus, marketing practitioners cannot achieve their economic and strategic goals using the copycat brand-far product category combination.

In the current study, we adopted an ERP-based method rather than traditional self-reporting tools to examine consumer preferences for copycat branded products. As a key neuroscience technology, ERP can markedly contribute to the systematic understanding of how consumers evaluate copycat branded products. First, using the ERP method, the present study captured not only people’s objective preferences for copycat brand-near products but also the underlying cognitive mechanism. That is, ERP revealed deep sources within the human brain that may explain why people show such preference behavior. We found two important neurological indicators, N400 and LPC, underlying the preference behavior, which described the brain’s multistage processing of information regarding copycat branded products. Here, N400 was suggested as a reflection of the categorization process, and LPC corresponded to the recollection process in working memory. Second, the neurological indicators demonstrated in this study could be applied as important references for assessing the effectiveness of copycat strategies for marketing researchers even before a copycat product exists. As elaborated in the introduction, ERP could capture more actual and accurate indications of consumers’ underlying preferences than traditional market studies based on self-reporting methods. Therefore, the effectiveness of a copycat product strategy could be tested according to the ERP indicators (N400 and LPC) observed here. Such assessments could help to efficiently allocate resources to develop products with market prospects, while indicating when to abandon products with low prospects in early stages of development. Moreover, if marketing researchers wish to improve the effectiveness of a copycat strategy, they may be able to adopt other combination strategies to affect the N400 and LPC responses.

The present study has two limitations. First, laboratory experiments inherently employ rigorous restrictions on experimental design, which may lack the richness of real-world settings. Further studies should address this issue by acquiring data in a more natural marketing environment. Second, our research only focuses on imitating the spelling features of leading brand names. Research has shown that half of the store brands in national US stores imitate leading brand packages in color or shape [46]. Further research should also investigate the evaluation of copycat brands and copycats that mimic other elements, such as perceptual similarity, logo design, and packaging.

## Conclusion

To conclude, the present study examined how consumers evaluate copycat branded products. An ERP approach was employed to explore the underlying neural mechanism of the evaluation process: categorization process and recollection process. We found that the copycat branded near product (CN) strategy was more preferred by people compared to normal branded near product (NN) and copycat branded far product (CF) strategies. In the cognitive process, this preference effect was separately reflected by a smaller N400 component and a larger LPC component in the CN condition compared to the NN and CF conditions. The N400 component was suggested to reflect the categorization process, while the LPC corresponded to the recollection process in memory. Generally, these findings imply that the copycat brand strategy will more effective when products are similar with the category of the leading brand, which offers important implications for marketing practices.

## Supporting information

S1 File The target stimuli list used in the experiment. The target stimuli include the brand names (original brand, copycat brand and control brand) and the product names (near product and far product) used in the experiment.
(DOCX)

S1 Table Behavioral and ERP data(N400 and LPC). The Behavioral data includes the mean acceptance rate. The N400 data includes the mean amplitude in the time window of 250 ms to 450 ms for four conditions (CN, CF, NN, NF) at F3, Fz, F4, FC3, FCz, FC4, C3, Cz and C4 electrodes. The LPC data includes the mean amplitude in the time window of 500 ms to 650 ms for four conditions (CN, CF, NN, NF) at C3, Cz, C4, CP3, CPz, CP4, P3, Pz and P4 electrodes.
(XLSX)
