# Supplementary material for: ERP evidence for consumer evaluation of copycat brands
Source: PLoS One. 2018 Feb 21;13(2):e0191475. doi: 10.1371/journal.pone.0191475 (PMC5842871; doi:10.1371/journal.pone.0191475)
Supplement: S1 File — The target stimuli include the brand names (original brand, copycat brand and control brand) and the product names (near product and far product) used in the experiment. (DOCX) [file pone.0191475.s001.docx]

Table 1 The target stimuli list used in the experiment

| **Original Brand** | **Copycat Brand** | **Control Brand** | **Near Product** | **Far Product** |
| --- | --- | --- | --- | --- |
| Coca Cola | Cock Cola | Feichan | ice tea | sushi |
| Pepsi | Pepshi | Fanta | juice | bread |
| Audi | Aoudi | Geely | motorcycle | battery |
| Benz | Benzi | Mazda | tyre | chair |
| Nike | Nika | Anta | leather shoes | mattress |
| Adidas | Adidass | Li-ning | suit | quilt |
| Reebok | Ruibuk | NewBalance | jeans | down jacket |
| Sony | Suony | Lg | ipad | automobile |
| McDonald | MikeDanlao | Kfc | turkey-burger | noodle |
| Nikon | Nicon | Canon | selfie stick | mobile phone |
| Samsung | Somsung | Apple | VR headset | furniture |
| Microsoft | Microsofia | Google | laptop | TV set |
| Philips | Fhilips | Gillette | aftershave | hair conditioner |
| Starbucks | Starducks | Costa | tea | soda |
| Lee | Li | Gap | sneaker | gym |
| Lay's | Leg’s | Pringles | bread | beef |
| Tide | Tige | Omo | laundry soap | bath foam |
| Toyota | Toyuda | Honda | bicycle | gasoline |
| Head & Shoulders | Heart & Shoulders | Dove | skin cream | laundry detergent |
| Crest | Krest | Colgate | dental clinic | shampoo |
| Wahaha | Wahaka | Nongfu | sandwich | vegetables |
| Huawei | Hwawei | Xiaomi | headphone | air conditioner |
| Boss | Bosses | Tommy | watch | jewelry |
| H&M | H&N | Zara | swimwear | carpet |
| Maotai | Maodai | Wuliangye | red wine | water |
| Dell | Dall | Hp | usb | cellphone |
